# Analytical Methods for Detection of Plant Metabolomes Changes in Response to Biotic and Abiotic Stresses

**DOI:** 10.3390/ijms20020379

**Published:** 2019-01-17

**Authors:** Anna Piasecka, Piotr Kachlicki, Maciej Stobiecki

**Affiliations:** 1 Institute of Bioorganic Chemistry, Polish Academy of Sciences, Noskowskiego 12/14, 61-704 Poznań, Poland; apiasecka@ibch.poznan.pl; 2 Institute of Plant Genetics, Polish Academy of Sciences, Strzeszynska 34, 60-479 Poznań, Poland

**Keywords:** biotic stress, abiotic stress, secondary, specialized metabolites, mass spectrometry, metabolome, metabolomics

## Abstract

Abiotic and biotic stresses are the main reasons of substantial crop yield losses worldwide. Research devoted to reveal mechanisms of plant reactions during their interactions with the environment are conducted on the level of genome, transcriptome, proteome, and metabolome. Data obtained during these studies would permit to define biochemical and physiological mechanisms of plant resistance or susceptibility to affecting factors/stresses. Metabolomics based on mass spectrometric techniques is an important part of research conducted in the direction of breeding new varieties of crop plants tolerant to the affecting stresses and possessing good agronomical features. Studies of this kind are carried out on model, crop and resurrection plants. Metabolites profiling yields large sets of data and due to this fact numerous advanced statistical and bioinformatic methods permitting to obtain qualitative and quantitative evaluation of the results have been developed. Moreover, advanced integration of metabolomics data with these obtained on other omics levels: genome, transcriptome and proteome should be carried out. Such a holistic approach would bring us closer to understanding biochemical and physiological processes of the cell and whole plant interacting with the environment and further apply these observations in successful breeding of stress tolerant or resistant crop plants.

## 1. Introduction

In the last few years climatic changes connected with an elevation of average temperature on the globe; an increase of greenhouse gases concentration in the atmosphere as well as elevation of environmental pollutions resulting from the human activity are observed. They result in changes in intensity of rainfalls in different regions of the world. These factors reduce the areas suitable for the sustainable agricultural production [1,2]. Both abiotic and biotic stresses have a detrimental impact on the crop plants cultivation and productivity. Due to the increasing human world population and growing food needs, introduction of new varieties of crop plants is the need of the moment. This demand is especially important in the areas endangered by the reduced water availability and increased salinity in agricultural areas or presence of pathogenic microorganisms or herbivores affecting the crop yield [3,4].

As a result of evolution processes plants elaborated different strategies for defense against biotic and abiotic stresses with which they have to cope during their vegetation. It should be underlined that due to the plants immovable life style their methods of defense against different stresses are to a high extent different from the strategies of animal reactions. The plant reaction to stresses is based on specific strategies that may be different for different plant species and may depend on the stress nature and severity. Primary and secondary metabolites synthesized in plants play various roles in response to stress: they regulate osmotic pressure within the cells, prevent to oxidation of cell components, deter herbivores, and prevent infection and growth of pathogenic microorganisms [5]. These compounds are produced as a result of diverse physiological and biochemical processes occurring in a plant ontogenesis, so it is important to reveal the respective metabolic pathways involved in plant functioning during the life cycle and their regulation. As crop plants are the basis of human food and animal feed, it is extremely significant to breed new efficient cultivars resistant against biotic and abiotic stresses and free from compounds that may induce anti-nutritional or toxic effects. The role of metabolic fingerprinting and metabolite profiling in assessment of unintended changes in metabolite composition caused by use of biotechnological approach to crop plant breeding has been recently presented [6]. Another field is devoted to biotechnological application of plants for synthesis of biologically active compounds that may be used as medicines, food additives, or cosmetics [7]. Also studies on environmental interactions of plants with the surroundings may be beneficial for better plant welfare, covering their growth and crop yield. Genetic and environmental perturbations are disclosed during metabolomics research. Applications of functional genomics techniques on genome, transcriptome, proteome, and metabolome levels may provide information about the structure and regulation/control of plant metabolic pathways functioning during the life cycle in various environmental conditions [7]. 

A brief overview of physicochemical methods applications for analysis of primary and secondary metabolites and utilization of specialized software will be given in this article.

The research conducted in the metabolomics field relays on two analytical methods: nuclear magnetic resonance (NMR) and mass spectrometry (MS) that are applied for identification, structural characterization, and quantitation of primary and secondary metabolites present in the biological material and further in the samples isolated as complex mixtures of natural products [8]. Hyphenation of mass spectrometric technics with chromatographic separation of natural products is the method of choice for this kind of analysis [7]. Various techniques that may be used in both combined analytical methods coupled in the hyphenated systems warrant relatively high sensitivity, repeatability, and selectivity. At present identification of all natural products in plant metabolome in a single experiment is not achievable. For this reason a method of analysis of large metabolomic data sets acquired by a consortium of several analytical laboratories using different analytical platforms was developed by bioinformaticists and biostatisticians to apply these data to validate metabolomics as a functional genomics tool [9]. The actual number of primary and secondary metabolites present in single plant species ranges from a few thousand to several dozen thousand and the total of currently known metabolite structures exceeds 200,000 and it may be even close to million [7,10]. These compounds occur in plant tissues in various concentrations differing by several orders of magnitude: from hormones present at femto- or picomolar levels to abundant sequestered compounds/natural products existing in the plant tissue in millimolar concentrations. Additionally, they substantially differ in their physicochemical properties, which influence their chromatographic behavior and possibility of identification dependent on mass spectrometric techniques applied. The problem of identification confidence has been approached by the Chemical Analysis Working Group of the Metabolomics Standards Initiative [11] that has defined 4 levels of compound identification. This topic has been recently reviewed [8,12].

Application of bioinformatic procedures for analysis of the obtained results permits comparison of metabolome data from different objects and further correlation with data from other omics level, such as information from transcriptome and genome and phenotype features registered for defined population of plant species or mutants selected for a single species grown under controlled conditions or stress [7]. This holistic approach resulted in the concept of systems biology basing on research conducted with different organisms: plants or animals. Conclusions drawn from studies conducted according to this methodology may allow designing more effective breeding strategies of new cultivars of crop plants showing better resistance against stresses and good quality of harvest or can be applied in elaboration biotechnological procedures for production of biologically active natural products [13].

## 2. Plants and Biotic and/or Abiotic Stress

**Figure 1:**
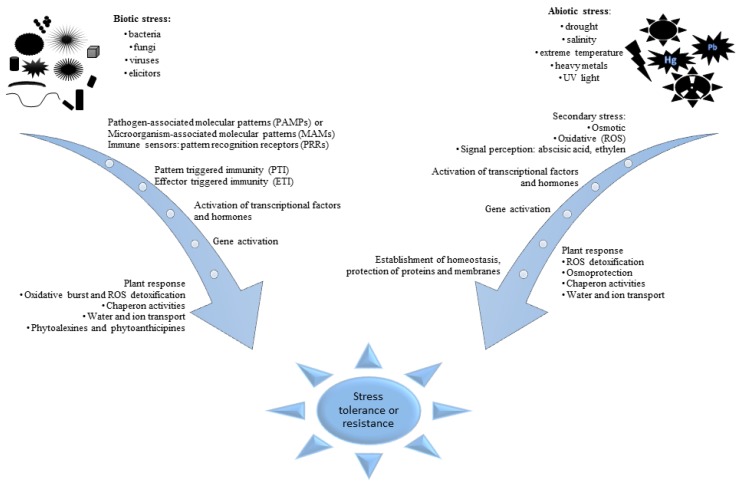
Stages of plant reactions to biotic and abiotic stresses. Particular processes can be specific to environmental stimuli. However, every of them leads to improvement of plant adaptation to stress and more effective response in future.

Plants growing in various ecosystems interact with the environment and they are exposed to various types of stress throughout the period of their life cycle. Stresses affecting plants may be divided into abiotic—caused by extreme temperatures, drought, salinity, UV radiation, wounding or heavy metals; and biotic—triggered by pathogenic microorganisms, nematodes, insects, or herbivores. The occurrence of both biotic and abiotic stresses has a large share in economic losses experienced by agriculture worldwide due to lower yields of harvests and decreasing area of arable land [14]. Mechanisms of plant reactions to the influencing stresses and defense against them have been intensively studied for many years. The studies are conducted with model and crop plants on different molecular levels: genome, transcriptome, proteome, and metabolome [7]. The results of the research carried out so far indicate that the reactions of plants to both kinds of stresses follow quite similar mechanisms. However, some principal differences between mechanisms of reaction on physiological and biochemical levels to biotic and abiotic stress were recognized on all molecular levels investigated. The basic differences are observed on initial stages of stress recognition (Figure 1). At the consecutive stages of plant response to different stresses, the induction of defense processes leads at all molecular levels to initiation of reactions which can lead to the restoration of homeostasis in surviving cells and tissue. Mechanisms of immunological response to invading microorganisms (viruses, bacteria, and fungi) were developed by plants during their evolution. The first stage of recognition of an invader presence is based on identification molecular structures, they are termed microbe- or pathogen-associated molecular patterns (MAMPs/PAMPs) [15,16,17,18,19,20]. Immune sensors of a different class, termed pattern recognition receptors (PRRs), activate a defense program: pattern-triggered immunity (PTI) or effector-triggered immunity (ETI). They are activated with PAMP, providing disease resistance in plants.

On the other hand, recognition of abiotic stresses occurrence and mechanisms of response to them are based on changes in physiological and biochemical processes initiated by these factors [21]. Phytohormones play an important role at the first stage of the abiotic stress recognition [22]. Oxidative stress signaling is initiated in plant tissues due to the increased abundance of signaling compounds, among others abscisic acid and ethylene. The plant response comprises initiation of intensive synthesis of compatible solutes such as proline, glycine, betaines, or sugars possessing osmolytic properties and compounds with strong antioxidative properties [23] and in effect the stomatal conductance is decreased [24]. The oxidative stress leads to activation of various processes starting after signaling pathways [25,26,27,28]. However it should be emphasized that plants respond to multiple stresses differently from how they react to single stresses. An abiotic stress can have reducing or enhancing effect on susceptibility to a biotic stress or vice versa [29].

Numerous metabolomic studies showing changes of various primary and/or secondary metabolites in crop plant tissues in response to stress have been published recently. As revealed using ^1^H NMR study of rice root metabolites, long-time low salinity stress caused enhanced accumulation of sucrose, allantoin and glutamate, whereas the levels of glutamine and alanine decreased [30]. Mass spectrometry based metabolomic studies showed that barley cultivars resistant to Fusarium head blight caused by *Fusarium graminearum* were characterized by increased amount of various phenylpropanoid metabolites, including flavonoids. These studies also revealed differences that were observed for resistance related metabolites suggesting possible biomarkers useful in marker assisted breeding [31,32]. Interestingly, several phenolic compounds, including phenylpropenoic acids derivatives and flavonoids, were demonstrated to be also induced in barley leaves in response to drought [33]. It was also shown that mild drought stress may cause significant increase of accumulation of secondary metabolites and thereby enhance the quality of plant-derived spices and fragrances of (for review see [34]).

Results of such studies were reviewed in plentiful articles concerned to applications of research carried out on model, crop or resurrection plants and performed for searching of biomarkers useful in breeding new varieties resistant to stresses were published in the last few years [35,36,37,38,39,40,41,42,43].

## 3. Analytical Methods Applied for Metabolome Composition Analysis and Description

In general, mass spectrometry (MS) is the method of choice for metabolites profiling in biological material [44]. There are several factors influencing the wide applicability of mass spectrometric techniques in plant sciences. These are: easiness of hyphenation of the mass spectrometers with separation methods (gas or liquid chromatography and capillary electrophoresis) and the possibility of application of different physical phenomena for ionization of the analyzed molecules and separation of created entities. This fact provides very good sensitivity, also reproducibility and versatility. An alternative approach to metabolome studies are methods based on nuclear magnetic resonance (NMR) [45,46,47]. However, they are characterized with a lower sensitivity but, on the other hand, quantitative comparisons of the analyzed compounds are straightforward. Due to these reasons both NMR and MS based methods may be used complementary [48]. The major advantages and shortcomings of these techniques are compared in Table 1.

The metabolomic analyses may be conducted in untargeted or targeted modes, depending on the aims of the performed studies. The untargeted approach is useful for metabolite profiling in order to monitor qualitative and quantitative changes of samples composition from biological material obtained from different environmental conditions or plants subjected to different stresses. In this case the sample preparation step is very simple and usually includes only extraction of biological material with proper organic or water-organic solvents. Mass spectrometric analyses may be performed with high resolution analyzers either using a direct infusion of the sample solution into the ionization chamber or performing chromatographic separation prior to the MS measurements [44,48]. In the case of targeted analyses samples may be enriched in a defined group of compounds using, for example, solid phase extraction (SPE) methods applying different solid phases in the utilized cartridges and various solvents for sample elution [49,50]. Reduction of the number of natural products present in the analyzed samples is beneficial from the viewpoint of achievable sensitivity in MS analyzer due to a lower ionization competition between the metabolites simultaneously present in the ion source and also the dynamic range of MS instruments. Interpretation of registered data is based on utilization of existing programs for identification and statistical interpretation of the results. Different mass spectrometric techniques may be applied during the targeted profiling of metabolites. Among others, structural characterization of natural products present in the analyzed mixture is possible. It is possible to elucidate different structural data about recognized compounds during a single analysis [51,52,53].

**Figure 2:**
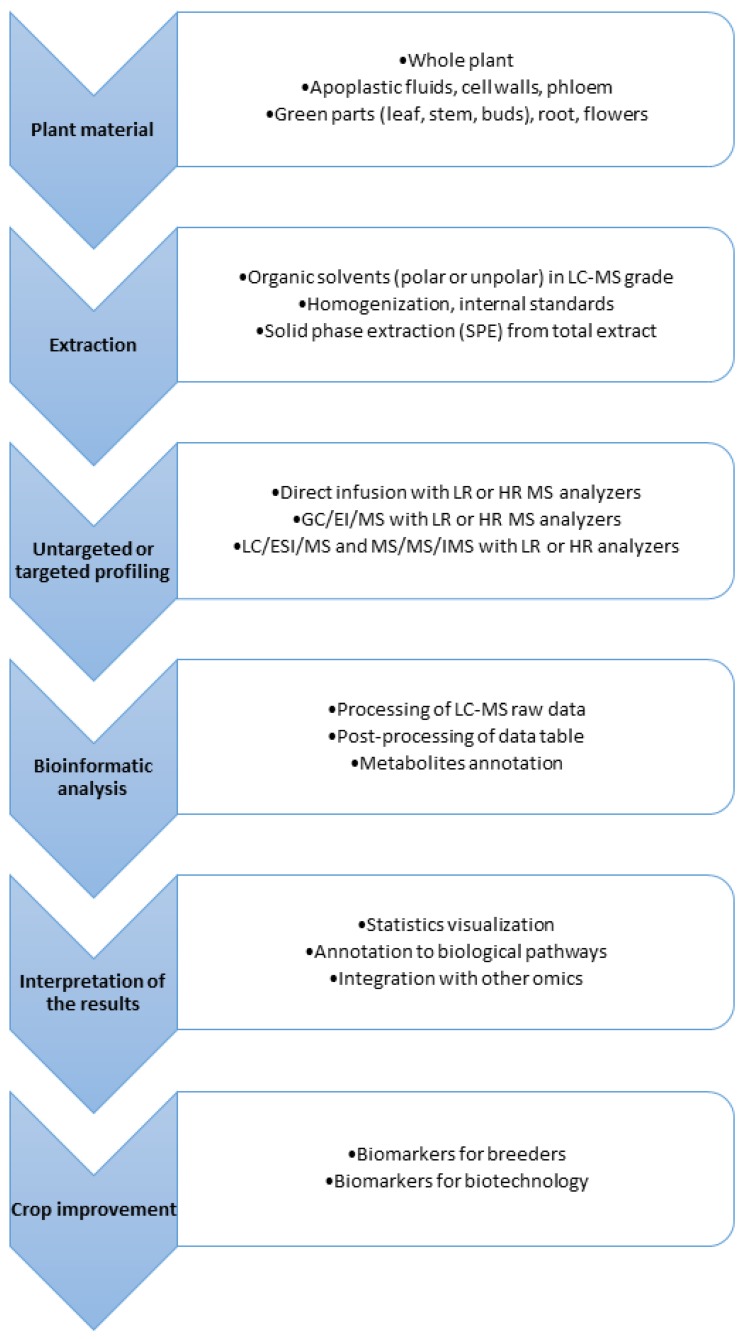
Mass spectrometric techniques in functional metabolomics flowchart—a useful tool for metabolome profiling and discovery alterations during abiotic and biotic stress. LR—low resolution mass spectrometers, HR—high resolution mass spectrometers.

It should be kept in mind that mass spectrometry is not a tool that allows gaining detailed identification and structural information about all analyzed natural products. In general, it gives information sufficient for tentative identification or annotation of the individual compounds. Mass spectrometers provide several methods to achieve this goal: elemental composition of the studied molecules may be calculated from the measurements of m/z values of ions with a high resolution and accuracy (with error below 5 ppm, and accuracy to the fourth decimal point); this annotation should be assisted by calculation of the isotopic pattern for the proposed protonated/deprotonated molecules the presence of different substituents and/or functional groups may be revealed from the fragmentation pattern obtained using collisional induced dissociation mass spectra (CID MS/MS). What more, chromatographic properties of the studied compounds should be taken into account during the annotation procedure. Some mass spectrometers provide also possibilities to separate ions of chromatographically co-eluting compounds using the ion mobility spectroscopy (IMS). However, it has been demonstrated that calculations of elemental composition of the ions recorded with a very high accuracy (with the errors of measurement below 1 ppm) is not sufficient for annotation of compounds with higher molecular weight abundant in the plant kingdom [54]. Additional information achieved during targeted metabolites profiling using CID MS/MS and especially ion mobility spectroscopy introduced in mass analyzers of some modern spectrometers warrants an increase of analyses selectivity [55] (Figure 2).

Another source of difficulties in interpretation of the recorded mass spectrometric data may be different ionization efficiency of compounds present in complex mixture due to their different physicochemical properties. This may result in various proton affinities or deprotonation efficiency during the electrospray (ESI) or atmospheric pressure chemical (APCI) ionization applied in the instrument. An additional problem is connected to the differences in compounds concentrations exceeding five orders of magnitude, when dynamic range of mass analyzers is five orders of magnitude. For example, plant hormones concentrations in extracted samples are in range lower than picomoles, and some natural products are in millimolar concentrations. Thus special strategies of targeted metabolites analysis have to be used [53].

Mass spectrometry can be applied also for imaging metabolomics, where arrangement of metabolites in the plant tissue or cell may be deciphered. Several techniques such as matrix assisted laser desorption ionization (MALDI) and desorption electrospray ionization mass spectrometry (DESI) are used for ionization of metabolites present in the samples [56,57,58].

## 4. Bioinformatics and Statistical Analysis in Metabolomics

Development of more efficient MS and LC techniques enables to run hundreds of metabolomic analyses in reasonable time. It is especially important for specialized metabolites analysis like glucosinolates, due to laborious sample preparation and complexity of plant extract [59]. The increasing sensitivity of MS devices influenced significantly the number of detected signals during each chromatographic run of studied samples and thus multiplied the size of obtained data files. This progress is particularly important in the field of plant metabolomics where thousands of possible specialized metabolites can be expected in an individual sample [60]. In addition, isotope-labeling for quantitative studies [61] or developed strategies called widely targeted metabolomics for fast secondary metabolites identification [62] cause creation of large LC-MS data sets. Selection of appropriate MS approaches and optimization of parameters for ion source and detector is only the first part of a long and difficult path for obtaining data of interest in the MS analysis (Figure 2). The biggest challenge in plant metabolomics is to extract information about specialized metabolites from the vast amounts of data produced by modern LC-MS systems [21]. In consequence, advanced statistical methods are necessary for comparison of all signals across all samples and quickly focus on relevant signals. Transformation of raw MS data into a numerical data matrix containing ordered, aligned and reduced information is necessary for statistics. Creation of data table demands dedicated tools for processing and alignment of raw MS data files. All of these steps from sample preparation, data acquisition, processing and statistical analysis as well as integration with data from other “omics” became an integral part of plant metabolomics.

### 4.1. Tools and Software Dedicated to Metabolomics

The current highly dynamic progress in sensitivity and selectivity of instruments applied in the LC-MS systems is accompanied by development of tools for processing of metabolomic data that are adapted to gathering large data sets from multi-sample experiments. Most of mass spectrometry producers offer commercial support in processing of raw data from their devices, for example programs: Sieve from ThermoFisher Scientific Inc., MetaboScape from Bruker Corp. Progenesis QI (Waters), MassHunter (Agilent) or MultiQuant (Sciex),) enable direct importing of files in formats generated by vendors’ LC-MS devices and processing followed by basic statistics in a simple and user friendly manner. However, a facilitated usage of this software and copyrighted methods of processing data may lead to unconscious errors in using the program and generating misleading results. The key to success in the metabolomic practice is the awareness of the principles of the processing steps and the purposefulness of all transformations on raw data prior to selection of appropriate parameters. The cost of licenses of this software is very often a limiting factor, especially in case of many potential users needing simultaneous access to the software. In addition, integration across different LC-MS platforms is impeded due to limitation of different format accessibility. 

Another solution is the usage of freely available tools. For users familiar with programming language open source scripts developed in R, Matlab, Python, Java etc. are available. The most often used and well known R-based command line interface software (CLI) is XCMS and its supports CAMERA [63]. It is available through the Bioconductor platform (www.bioconductor.org) and offers a broad spectrum of functions from the raw data transformation through peaks detection to statistical analysis. Mummichog is a set of scripts implemented in Phython for analyzing data from high throughput, untargeted metabolomics [64]. Matlab-based scripts are also widely used [65]. OpenMS provides methods for metabolomic workflow by C++ with binding to Phyton. The ability to handling the procedures using the command line interface (CLI) provides opportunities to test and tune variety of parameters and to suit the processing to own experiments. Nevertheless, persons interested in plants metabolomics are mainly biologists and crop breeders who are not experienced with programming environments such as MATLAB or R and most often there is the necessity of their cooperation with bioinformaticians.

XCMS Online (https://xcmsonline.scripps.edu) based on XCMS scripts is the online service which enables direct uploading of raw data and guides the user through the comprehensive workflow of data processing and statistical analysis [66], default parameter sets for different instrument setups (e.g., predefined set up for HPLC/Orbitrap or UPLC/Orbitrap) can be selected to facilitate the usage. Uploading of raw data and processing, annotation of signals to Metlin database, useful for plant specialized metabolite and storage of projects is available after login creation. This online service was used in many plant stress metabolomics studies [67,68]. Uploading raw data through internet especially from more complex plant metabolomic experiments which can contain more than hundred data files is highly time consuming. A limited space for data storage in the XCMS servers is also problematic. Recently possibilities have been developed for automatic file transfer from the LC-MS system directly after the data acquisition to XCMS online by a data streaming platform, XCMS Stream, that increases the efficiency of the online server use and shortens time necessary for data processing [69]. MeltDB (https://meltdb.cebitec.uni-bielefeld.de) is also a suitable tool for plant metabolomics processing, integration with genomic results and statistical analysis [70]. Alternatively, Workflow4Metabolomics (W4M, http://workflow4metabolomics.org) is a web-based platform developed in a Galaxy environment including functions for processing, statistics and annotation of metabolomics data [71]. It is also possible to run W4M by virtual machine for a local installation. For the overview of the available tools see Table 2.

Other tools for processing raw LC-MS data can be installed on the users’ computers. Most of the popular tools like MZmine 2 [76] and MzMatch [80] work using the Java Technology. MetAlign [77], MS-Dial [78] and the recently developed iMet-Q (intelligent Metabolomic Quantitation) [84] work by Windows graphical user interfaces (GUI) and do not require additional installation. The most popular software are programs based on XCMS scripts in GUI which can be used without programming experience, for example MzMatch or MZmine2 (for a review of the available software see [91]. TracMass 2 is a similar software based on Matlab scripts [86] and eMZed is an open source framework based on Phyton. However, some functionalities from XCMS are also available [79].

MaxQuant [87], OpenMS [88] and ProtMAX [89] that were software originally dedicated to proteomics can be also implemented for metabolomic data processing. OpenMS was originally designed for proteomics, however, it now also includes the FeatureFinderMetabo [92] module, specifically designed for non-targeted metabolomic data.

**Figure 3:**
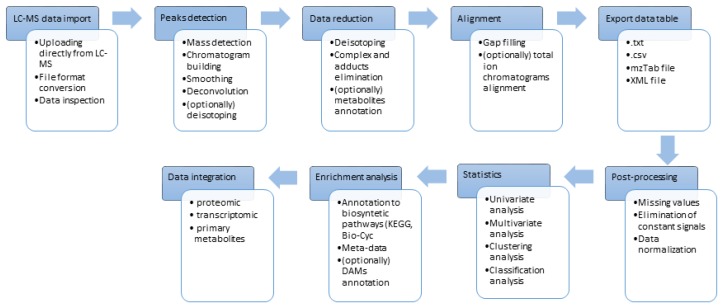
Scheme of workflow in metabolomics data processing, post-processing and statistics in untargeted metabolomics. Some of the steps can be shifted depending on chosen bioinformatic method. In some software’s alignment procedure can be conducted at beginning of workflow as well as deisotoping can be incorporated into peak picking. Annotation of metabolites can be performed after alignment on entire data table or can be focused on differentially accumulated metabolites (DAMs) of interest.

These above-mentioned programs enable processing of the metabolomic raw data by multistep flowchart (Figure 3). Each software package applies a different strategy of processing and each basic algorithm for particular processes requires entering the right parameters. It is extremely important to get acquainted with the rules of following algorithms and overall strategies for processing before starting the analysis. Every step of processing and every parameter is equally important to get reliable and accurate results.

The first problem to overcome with processing of raw LC-MS data is the conversion of data format generated by LC-MS systems. Acquisition of data is carried out in vendors’ specific format, sometimes in complex folders of files. Not all of them can be implemented to particular software. Then it has to be converted to open access common data formats such as netCDF (Network Common Data Form), mzXML, or mzML which are utilized by all software and easily adapted for processing transformations. Most of the LC-MS systems producers provide conversion functions in the delivered programs. However, if necessary, MSConvert from ProteoWizard package can be used as a multifunctional and a comprehensive converter [93]. Specific processing tools, for example MS-Dial, dedicated especially for independent MS/MS data processing need a conversion of raw data into ABF (analysis base file) by a provided converter.

### 4.2. Peak Picking

Importing data in a proper format starts the processing workflow. Every step can be conducted in different strategies depending on the chosen tool. The first step is usually peak picking that includes algorithms for Mass detection followed by Chromatogram Building and Peak Deconvolution.

Mass detection requires raw data specification (mass resolution, mass precision, peak shape, and noise). It is necessary to define the acquisition mode: centroid or profile (continuous) data in m/z for choosing the optimal algorithm for the signal detection. Most programs, iMet-Q, MS-Dial and MZmine2 perform the centroiding process firstly. The GridMass method implemented in MZmine2 is dedicated to fast and accurate detection of LC-MS peaks [94]. The algorithm detects the position and boundaries of masses, searches for a local maximum of intensity for certain m/z which are defined as features. The method was proved to be useful for complex plant metabolomics [76] and lipidomics [95].

Mass Detection is the first line of reducing data size by removing of noisy signals (false positives). Defining the baseline cut-off level prevents the detection of parts of chromatograms close to the baseline level. The remaining peaks that stand out above the baseline level are recognized if they fulfill the height and duration requirements. iMet-Q supports an expanded method of noise removing in relation to an average intensity of the borderline in a defined number of scans.

Mass lists generated in all MS scans serve for building chromatograms for defined m/z range which should be set manually depending on the raw data resolution. The chromatograms have to be subsequently smoothed and decomposed (deconvoluted) into unique peaks. As in each chromatogram diverse peaks characterized by a low and high signal-to-noise ratio levels as well as broad peak shapes can be observed simultaneously, an appropriate method of deconvolution should be selected carefully and adapted to every LC-MS set individually. Several algorithms for chromatograms building and deconvolution are available. Among them, centWave developed for XCMS, is most often used [96]. The process starts with finding a highly dense region of m/z within a specific mass error (regions of interest, ROIs) across all scans. Next, ion chromatograms based on m/z intensities for each ROI are created. The extracted ion chromatograms that display a peak shape are then added to the peak lists. centWave as an efficient algorithm for high-resolution complex data and it became the basic one and used routinely to detect specialized plant metabolites (only to mention a few applications: [97,98,99]. An extension and complement of centWave is the Warpgroup method for increasing real peaks detection [100]. This approach has been subsequently implemented into the XCMS Online, and has been also used by several other programs: eMZed, Mzmatch, MZmine2, and with some modifications, in MET-COFEA [82].

MZmine2 offers the largest assortment of peak detection methods capable for optimizing this procedure. Among several methods centWave XCMS is available as well as developed for MZmine2 ADAP wavelet which is an expansion of the centWave algorithm [101].

An unique approach to peak picking (called peak spotting) dedicated to the MS/MS data is offered by MS-Dial in which peak detection algorithm is applied to the base-peak chromatogram for detection of “spots” in two defined continuous data axes: retention-time and accurate mass [78]. Each spot can be then deconvoluted by MS2Dec procedure. The algorithm first extracts the product spectra for each precursor peak on all MS/MS chromatograms to recover the precursor-product links. This method has been recently implemented into plant stress metabolomic for automatic untargeted metabolome profiling and identification of specialized metabolites [102,103]. FeatureFinderMetabo is an extension of the OpenMS framework adapting this proteomic platform for metabolomics. It comprises two main stages, the mass trace detection and feature assembly based on building model scoring for mass deviation and isotope profile. It can be applied to specialized metabolites as well as peptide and lipids analyses [92].

Selection of the appropriate parameters in the peak picking phase is essential for a proper peaks determination. However, it is difficult to optimize the algorithm, especially for plant metabolomics due to the high diversity in signal intensity and shape. Recently, a R-based software package IPO (Isotopologue Parameter Optimization) has been developed to optimize XCMS peak picking parameters by using natural, stable 13C isotopic peaks to calculate a peak picking score from different LC-MS instruments [104].

### 4.3. Data Reduction

After defining peaks in all samples, reduction of data size and normalization can be optionally implemented. Deisotoping algorithms recognize the isotopic clusters of the same metabolites and then remove isotope bands occurring in a smaller amount, leaving the most intense one. As was mentioned above, deisotoping is incorporated into feature assembly related to peak picking in FeatureFinderMetabo. An extension of XCMS and CAMERA for an enhanced isotope cluster detection and validation has been recently developed [105] and used in plant stress metabolomics [106,107]. The deisotoping step is crucial in case of sulphur containing metabolites for which detection is based on mass shift between isotopes 32S (the mainly occurring) and 34S (occurring at a low intensity). Differences in the exact mass and signal intensity between isotopes can be detected by the peak picking procedures. Nevertheless, deisotoping procedures in metabolomic studies can overlook these ions and make it difficult to identify them later [108]. A further data reduction can implement filtering off duplicated peaks, adducts and multiply charged molecules or removing non important signals defined by the user. Artificial and noisy peaks should be also removed. The data reduction processes aim to decrease the complexity of data and speed up the data interpretation. XCMS and CAMERA offer automatic data reduction cooperating with signals annotation to online databases. In addition, supporting tools for XCMS and CAMERA were also developed [109].

### 4.4. Data Set Alignment

Next steps aim to combine all information across the sample set into one data table. A major problem with the alignment is related to the retention time shifts for a single metabolite among samples due to analytical conditions. On the other hand, many detected features with very low differences in their respective m/z and retention time may be incorrectly aligned. Two main strategies in metabolomic alignment are used. The first is based on the total ion chromatogram alignment by iterative time correction towards a reference chromatogram. There are several methods that introduce alignment at the beginning of data processing, before the peaks picking. Algorithms supporting these methods can be also used for the UV-based chromatographic data processing in plant stress metabolomics [33]. Among them correlation-optimized warping (COW) [110], dynamic time warping (DTW) [111] and their modifications are commonly used. The second approach of alignment requires defined peaks with the assigned retention times. XCMS uses this strategy in OBIWARP algorithm [112]. It shifts peaks within all samples according to a reference sample. Then the correction curve is applied in order to group samples. An extension of this method is the alignment based on the random sample consensus (RANSAC) in MZmine2 dedicated to cope with a non-linear deviation of the retention times among samples by iterative estimation of the most fitted model [76]. The method has been proven to be suitable to plant stress studies in which outliers are frequent [113]. Joint aligner developed for MZmine2 and implemented also in MS-Dial were used in plant metabolomics [103,114])

An extended method for alignment data with larger retention time drift and MS/MS spectrum was described recently [115]. Zhang and co-workers [116] have introduced MET-XAlign, compound-based alignment which operates on the deduced molecular mass and estimated retention time and it postulates possibilities to aggregate meta-data and different electrospray ionization modes. 

### 4.5. Post-Processing and Statistic of Data Table

Aligned signals can be exported in several formats (e.g., .txt, .csv, mzTab) to further statistical analysis. Some statistical functions are available in the processing software. Metabolomics statistical tools can be downloaded or are available online in the user friendly GUI. The tools enable an enhanced statistics in a variety of experimental setups in order to decipher important biological information. The obtained data table must be properly prepared (post-processed) prior to the statistical analysis. Most of these necessary calculations can be executed in a workflow of statistical software. Specialized metabolites can be synthesized de novo in response to the stress conditions, thus in data table for certain signals control samples after alignment problematic missing values will be observed for part of samples. The missing values are problematic from the statistic point of view and methods for handling them can significantly affect the subsequent data analyses and interpretations. Several strategies were implemented for handling missing values, such as replacement by a mean/median, k-nearest neighbors, random forest, probabilistic PCA, Bayesian PCA, singular value decomposition (SVD) imputation (for a review see [117]). Recently a new method based on support vector regression from machine learning has been developed [118]. On the other hand, missing values appears also due to omissions in the previous processing steps. Peaks with irregular peak shape or peaks close to the detection limit may be missed in some samples whereas in others they could be explicated. For this case MZmine2 includes two possibilities of missing values imputations: Gap filling by Peak Finder as well as Same RT and m/z range. Both are based on re-search of chromatograms under the cut-off parameters defined by user that finally results in a reconstructed data table.

However, problem with the missing values or zero value can be solved by simply filling all gaps with defined small values as was proposed in MetaboAnalyst [73]. It is comprehensive online statistical platform for metabolomics data interpretation and integration with other “omics”. MetaboAnalyst offers several methods for data filtering: removal of very small signals, values near constant across all samples or signals with low repeatability in comparison to quality control samples. Differences in specific metabolites concentration within or between samples can be caused by biological variations. Thus, normalization of data should be conducted for a better comparison of biological signals [119]. Normalization among samples can be achieved by dividing the signal intensities by their sum, or a standard compound. Scaling and logarithmic transformation should be used for minimizing the effect of the variation within a particular biological sample. 

### 4.6. Visualization of Statistical Results

Statistical elaboration of dense data matrices is a bottleneck of metabolomics. The goal is to identify the metabolites that are significantly changing between classes of biological samples. Many statistical approaches were commonly implemented in metabolomics and reviewed elsewhere [119,120]. Most of the used methods were previously implemented in transcriptomic or proteomic high-throughput data analysis. The most clear classification can divide the methods on univariate (Fold Change Analysis, *t*-tests, Volcano Plot), and multivariate (Principal Component Analysis, PCA; Partial Least Squares—Discriminant Analysis, PLS-DA). Both of these approaches are complementary for the metabolomic studies. 

Univariate statistic methods are frequently used to reduce a large number of detected signals and indicate the differentially accumulated metabolites. The choice of the proper strategies for this purpose depends on the type of data. For data close to normal distribution, parametric tests like the *t*-test and one-way ANOVA can be applied. The distribution can be achieved by a logarithmic transformation. If data are still far from the normal distribution, non-parametric approaches like Mann–Whitney U-test or the Kruskal–Wallis test should be implemented. These methods do not use the intensity of signals to compare the groups, but rather the rank of these values. The most useful univariate test is ANOVA which measures the ratio of the variation between the groups to the variation within the groups. As an output a p-value is calculated, which corresponds to the probability of finding the observed test-statistic, under the assumption that the groups where not different. In case of complex metabolomic data matrices it is necessary to apply a modification of p-value to reduce the number of false positives. Among them multiple testing corrections: Bonferroni correction and Benjamini–Hochberg false discovery rate procedure (FDR) are most often used. ANOVA together with Fold Change analysis serve for creation of volcano plots, which are an useful approach for extraction and visualization of differentially accumulated metabolites in two dimensional space of statistical importance (*p*-value or FDR) and biological importance (fold change).

Multivariate analysis methods are suitable for reduction of high-dimensionality in metabolomic data tables. They show not only changes of single metabolites between different groups, but also the dependency structures across samples. PCA is very often the first step of explorative data analysis due to generation of easily interpretable plots. PLS-DA (discriminant analysis) regression, which belongs to the class of supervised models, distinguishes metabolite profiles that are strongly associated with the predefined groups.

Other unsupervised multivariate techniques, such as clustering analysis, tend to group and visualize samples according to intrinsic similarities. Among them the hierarchical cluster analysis (HCA) is widely used for chemotaxonomy [121,122]. Self-organizing map (SOM) and k-means clustering can serve for construction of heatmaps for visualization of signal intensity distribution across all samples.

A machine learning method Support Vector Machine (SVM), primarily widely used in genome studies, was also implemented in metabolomics for supervised signal classification and biomarkers visualization in untargeted studies [123]. This method and the Random Forest method, suitable to metabolomic feature selection as well as for outliers selection in statistical workflows, were implemented in MetaboAnalyst software.

### 4.7. Metabolomics Enhancement

Progress in metabolomics resulted in generation of multifunctional metabolomic web-based services. They offer not only processing of raw data but also integration with proteomic and transcriptomic results. In addition, they enable enrichment analysis and annotation of metabolites directly onto biosynthetic pathway and facilitate searching for significant metabolites. XCMS Online, originally a data-processing platform, nowadays has become multifunctional, the most often used tool in metabolomics [65]. It enables annotation of metabolites to Metlin database and variety of statistical and integrational functions. PRIMe is a web-based service for metabolomics and transcriptomics [72]. Available for downloading software from PRIMe cover most of aspects of metabolomics. PRIMe also includes a wide metabolomics data base with results from different experiments. MetaboAnalyst is dedicated to statistics for targeted or untargeted metabolomic data sets. It enables general statistics, biomarker analysis, two-factor/time-series analysis and power analysis). In addition, it facilitates integration to other “omics” by biomarker meta-analysis, joint pathway analysis, and network explorer module. Workflow4metabolomics (W4M) also serves for the comprehensive post-processing of metabolomic data table with batch correction and enhanced visualization of statistical results as well as broad possibilities of searching databases and metabolites annotation [71]. 

Specialized tools provide solutions to automatic metabolites identification. Annotation of metabolites based on their fragmentation scheme can be supported by specialized tools like MetFrag [75] or MS-Finder [90]. The later one can be linked directly with the MS-Dial from the PRIMe platform. These programs enable formula predictions, possible metabolites annotations and structure elucidations. The commonly searched data bases are KEGG, Bio-CYC, PubChem, and ChemSpider. However, precise determination and identification of the metabolite being tested is impossible by means of mass spectrometry. Searching across public databases usually gives a few to even a hundred possible matches, so an exact determination of metabolite is imposed. Additional strategies should be added to boost the compound identification step.

A different type of metabolomic tools serves for extracting of significantly differentiated metabolites from the data set. One of example can be the MarVis toolbox, which is dedicated to metabolomic and transcriptomic marker visualization [85]. 

Prepared data table can be imported to MarVis for annotation of metabolites and cross-omics integration. The analysis is supported by an extensive framework for pathway enrichment from KEGG and Bio-Cyc and meta-analysis. This tool is promising for integration of metabolomics with transcriptomic data in plant studies [124].

In spite of many possibilities in processing and statistical analysis of metabolomics data it is very important to have some criticism on automatically done metabolomic workflow. Re-inspecting of obtained results should be obligatory. The best way will be implementation of another independent tool for the same study and comparison the results. Confirmation of the obtained results may exclude some speculative achievements in metabolomic workflows. Choosing the right validation methods step is crucial to achieve reliable results. 

Analysis of plant metabolome in stress conditions is a challenge and the large diversity in the metabolomic profiles between treatments demands careful selection of LC-MS parameters. The metabolomic experiments are usually very complex and due to this it is demanded to apply enhanced statistics and visualization methods, very often going beyond the possibilities available via metabolomics tools. A significant progress should be made in accurate and effective identification and quantification of metabolites. 

Integration of metabolomics results with other levels from the systems biology is necessary for detailed understanding of the molecular mechanisms underlying plant metabolism during stress [125,126] and metabolic variations in plants [127]. Integration methods are desired for comprehensive understanding biological processes in plant [128]. Rapid and easy integration with other omics is still limited due to fragmentary resources in databases and troubles with defining relational models of relationships between omics [129]. Nevertheless, it is worth to develop new effective tools for deeper metabolomic investigation in multivariate and integrative manner. Characterization of molecular biomarkers linked to certain biotic or abiotic stresses is highly desired by plant breeders for developing crops with increased resistance and increased yield production. The aim is to capture not only the subtle changes of a single metabolite or gene level, but also primarily the interconnectivity between the molecular components given the complexity of biological systems. 

## Figures and Tables

**Table 1:** Limitations and benefits of nuclear magnetic resonance and mass spectrometric instruments in metabolomic studies.

Instrumental Method	Selectivity *	Sensitivity	Quantitative Analysis	Drawbacks	Additional Comments
NMR	Good	Low	Good	Low number of identified compounds	Lack of tools for bioinformatic analysis
LC/NMR	High	Low	Good	High cost of analyses, low separation of LC column	Absolute structural characterization possible
Direct infusion MS	Low	Good	Acceptable	Ionization competition between compounds	Possible estimation of elemental composition of protonated molecules with high resolution mass analyzers
GC/MS ^a^ or GC/GC/MS ^a^	Good	High	Acceptable	Need of derivatization, low molecular mass range only up to 500 Da	Good separation of compounds with GC column, improved with the use of the GC/GC technique
GC/MS/MS ^a^	Good	High	Good	As above	As above
LC/MS ^a^	Good	High	Acceptable	Low separation of LC column	Possible estimation of elemental composition of protonated molecules with high resolution mass analyzers
LC/MS/MS ^a^	Good	High	Good	Low separation of LC column	Possible estimation of elemental composition of protonated molecules with high resolution mass analyzers, possible differentiation of isomeric and isobaric compounds
CE/MS ^a^	Good	Very high	Acceptable	Difficulties of stable hyphenation of CE instrument with mass spectrometer	Good separation of compounds with CE instruments

* possibilities of structural characterization; ^a^ possible application of low- or high resolution of mass analyzers.

**Table 2:** Summary of functions on freely available metabolomics tools with graphical user interface for comprehensive workflow starting from data processing, post-processing, statistics, integration with other omics and metabolite annotation.

Tool	Data Processing	Data Post-Processing	Statistical Analysis	Integration With Other Omics	Annotation to Metabolomics Databases	Annotation to Pathways Databases	References
online services							
XCMS online	✓	✓	✓	✓	✓	✓	[66] https://xcmsonline.scripps.edu
PRIMe	✓	✓	✓	✓	✓	✓	[72] http://prime.psc.riken.jp/
MeltDB	✓	✓	✓	✓	✓	✓	[70] https://meltdb.cebitec.uni-bielefeld.de
Workflow4Metabolomics (W4M)	✓	✓	✓	✓		✓	[71] http://workflow4metabolomics.org
MetaboAnalyst		✓	✓	✓	✓	✓	[73] https://www.metaboanalyst.ca/
Metabox		✓	✓	✓	✓	✓	[74]
MetFrag					✓	✓	[75] http://c-ruttkies.github.io/MetFrag
local installation							
MZmine2	✓	✓	✓		✓	✓	[76]
MetAlign	✓	✓	✓		✓		[77]
MS-Dial	✓	✓	✓				[78]
eMZed	✓	✓	✓		✓		[79]
MzMatch	✓	✓	✓		✓		[80]
IDEOM	✓	✓	✓		✓		[81]
MET-COFEA	✓	✓	✓		✓		[82]
MAVEN	✓	✓	✓				[83]
iMet-Q	✓	✓	✓				[84]
MarVis		✓	✓	✓		✓	[85]
TracMass 2	✓						[86]
MaxQuant	✓	✓	✓		✓		[87]
OpenMS	✓	✓	✓		✓		[88]
ProtMAX	✓	✓	✓		✓		[89]
MS-Finder					✓	✓	[90]
